# Correction to “Efficacy of Dual‐frequency Noninvasive Monopolar Radiofrequency in Skin Tightening: Histological Evidence”

**DOI:** 10.1111/srt.70335

**Published:** 2026-02-26

J. Hong, H. G. Ryu, C. Park, et al., “Efficacy of Dual‐Frequency Noninvasive Monopolar Radiofrequency in Skin Tightening: Histological Evidence,” *Skin Research and Technology* 30, no. 6 (2024): e13821, https://doi.org/10.1111/srt.13821.

The Conflict of Interest Statement is corrected from:

“The authors have no conflict of interests to declare.”

to:

“J.H., H.G.R., C.P., J.P., K.K., K.M.M.L., and S.I.C. are employees of Cynosure Lutronic, the manufacturer of the monopolar radiofrequency device evaluated in this study. The study was conducted as an exploratory preclinical investigation. Cynosure Lutronic provided the monopolar radiofrequency system used in this study and had no role in the study design, data collection, data analysis, interpretation of the results, or decision to publish.”

The authors apologize for this error and for the inconvenience it may have caused.

